# Brain development of a school-aged boy with autism spectrum condition talented in arithmetic: a case report

**DOI:** 10.1093/psyrad/kkae008

**Published:** 2024-04-18

**Authors:** Weixing Zhao, Lei Li, Xiujie Yang, Xiaotian Wang, Juan Kou, Jia Chen, Huafu Chen, Qi Wang, Xujun Duan

**Affiliations:** The Clinical Hospital of Chengdu Brain Science Institute, School of Life Science and Technology, University of Electronic Science and Technology of China, Chengdu 610054, PR China; MOE Key Laboratory for Neuro information, High-Field Magnetic Resonance Brain Imaging Key Laboratory of Sichuan Province, University of Electronic Science and Technology of China, Chengdu 610054, PR China; The Clinical Hospital of Chengdu Brain Science Institute, School of Life Science and Technology, University of Electronic Science and Technology of China, Chengdu 610054, PR China; MOE Key Laboratory for Neuro information, High-Field Magnetic Resonance Brain Imaging Key Laboratory of Sichuan Province, University of Electronic Science and Technology of China, Chengdu 610054, PR China; Faculty of Psychology, Beijing Normal University, Beijing 100875, China; The Clinical Hospital of Chengdu Brain Science Institute, School of Life Science and Technology, University of Electronic Science and Technology of China, Chengdu 610054, PR China; MOE Key Laboratory for Neuro information, High-Field Magnetic Resonance Brain Imaging Key Laboratory of Sichuan Province, University of Electronic Science and Technology of China, Chengdu 610054, PR China; Institute of Brain and Psychological Sciences, Sichuan Normal University, Chengdu 610066, China; Department of Developmental-Behavioral Pediatrics, The Affiliated Mianyang Hospital, School of Medicine, University of Electronic Science and Technology of China, Chengdu 610054, PR China; The Clinical Hospital of Chengdu Brain Science Institute, School of Life Science and Technology, University of Electronic Science and Technology of China, Chengdu 610054, PR China; MOE Key Laboratory for Neuro information, High-Field Magnetic Resonance Brain Imaging Key Laboratory of Sichuan Province, University of Electronic Science and Technology of China, Chengdu 610054, PR China; Department of Developmental-Behavioral Pediatrics, The Affiliated Mianyang Hospital, School of Medicine, University of Electronic Science and Technology of China, Chengdu 610054, PR China; The Clinical Hospital of Chengdu Brain Science Institute, School of Life Science and Technology, University of Electronic Science and Technology of China, Chengdu 610054, PR China; MOE Key Laboratory for Neuro information, High-Field Magnetic Resonance Brain Imaging Key Laboratory of Sichuan Province, University of Electronic Science and Technology of China, Chengdu 610054, PR China

**Keywords:** Autism spectrum condition, arithmetic talent, gray matter, voxel-based morphometry, magnetic resonance imaging, case report

## Abstract

Whereas autism spectrum condition is known for its social and communicative challenges, some autistic children demonstrate unusual islets of abilities including those related to mathematics, the neurobiological underpinnings of which are increasingly becoming the focus of research. Here we describe an 8-year-old autistic boy with intellectual and language challenges, yet exceptional arithmetic ability. He can perform verbal-based multiplication of three- and even four-digit numbers within 20 seconds. To gain insights into the neural basis of his talent, we investigated the gray matter in the child's brain in comparison to typical development, applying voxel-based morphometry to magnetic resonance imaging data. The case exhibited reduced gray matter volume in regions associated with arithmetic, which may suggest an accelerated development of brain regions with arithmetic compared to typically developing individuals: potentially a key factor contributing to his exceptional talent. Taken together, this case report describes an example of the neurodiversity of autism. Our research provides valuable insights into the potential neural basis of exceptional arithmetic abilities in individuals with the autism spectrum and its potential contribution to depicting the diversity and complexity of autism.

## Introduction

Autism spectrum condition (ASC) encompasses a group of neurodevelopmental conditions characterized by challenges in social interaction and communication, as well as the presence of repetitive behaviors or a narrow range of interests (American Psychiatric Association, 2013). Individuals with ASC typically demonstrate diverse intellectual and language development, leading to significant variations in cognitive profiles (Munson *et al*., 2008). However, they also possess a unique way of processing information. This unique processing style is not always indicative of disability and can even manifest as areas of exceptional talent (Baron-Cohen & Lombardo, 2022). Although robust epidemiological data are lacking, a few studies suggest that some individuals with ASC demonstrate exceptional abilities in specific domains, often in domains such as music, visuospatial skills, art, and arithmetic. These abilities can be notably superior to their other skills and may even be considered absolute strengths compared to their peers (Miller, 1999; Mottron *et al*., 2006; Baron-Cohen & Lombardo, 2022).

Numerous theories have been proposed to explain the exceptional abilities observed in individuals with ASC, including Weak Central Coherence, Enhanced Perceptual Functioning, and hyper-systematizing theories (Happé & Frith, 2006; Baron-Cohen *et al*., 2009; Mottron *et al*., 2009). These theories concentrate on the hyperattention to detail and hypersensitivity to sensory stimuli in ASC. Proponents of the hyper-systematizing theory have demonstrated the significance of the lateral frontoparietal regions and sensory and perceptual systems in the unique cognitive processes observed in ASC, suggesting a potential neural mechanism for the talent observed in ASC (Baron-Cohen & Lombardo, 2022). Brain imaging studies revealed that arithmetic engages a large network of interconnected brain regions including the dorsolateral prefrontal cortex, ventrolateral prefrontal cortex, posterior parietal, occipito-temporal, anterior cingulate, and hippocampal areas (Peters & De Smedt, 2018). Voxel-based morphometry (VBM) is a neuroimaging technique for analyzing regional differences in brain tissue volume and density (Ashburner & Friston, 2000). VBM investigations have reported significant positive associations between gray matter in the left intraparietal sulcus and right hippocampus and arithmetic abilities in typically developing children (Li *et al*., 2013; Price *et al*., 2016). Conversely, structural imaging investigations conducted on children with arithmetic difficulties have shown a reduction in gray matter within critical regions of the arithmetic network, including the inferior frontal gyrus, posterior parietal cortex, parahippocampal gyrus, and occipito-temporal cortex (Rotzer *et al*., 2008; Rykhlevskaia *et al*., 2009; Han *et al*., 2013; Ranpura *et al*., 2013). These findings provide preliminary insights into the possible complex relationship between arithmetic ability and brain structure.

In this report, we presented a case of a school-aged boy with ASC who exhibited extraordinary arithmetic ability. We obtained structural magnetic resonance imaging (MRI) scans of the case and conducted an extensive battery of behavioral assessments. We performed a gray matter analysis based on VBM to investigate the features of the arithmetic-related brain regions in the case and whether these features differed from those of typically developing controls (TDCs). We hypothesized that the arithmetic-related brain regions in the case may have distinct structural characteristics from those of TDCs, and that these structural differences may be the source of his extraordinary arithmetic ability.

## Case presentation

### Referral information

The case was focused on a boy of 8 years and 11 months of age, diagnosed with ASC, who showed remarkable arithmetic skills. He has been diagnosed with hearing impairment in his right ear, speech delay, attention deficit and hyperactivity disorder, and mental retardation (detailed information is provided in Table 1). According to his parents, he demonstrated remarkable arithmetic skills in first grade. He could perform calculations involving two-digit addition, subtraction, multiplication, and division within seconds, and he could complete multiplication calculations with three- and four-digit numbers in 20 seconds. Furthermore, he demonstrated a heightened sensitivity to time and distance; he could provide the current time based on the position of the Sun within a 2-minute margin of error, and he could estimate the distance traveled.

**Table 1:** Clinical: symptoms and development by age.

Symptom	Age (years)
Mental retardation	2
Right ear hearing impairment	2
Significant speech delay	3
ADHD diagnosis	7–8
Significant speech delay (language skills were between 3.5 and 5 years)	7–8
Mental retardation	7–8

ADHD: attention deficit and hyperactivity disorder.

### Clinical symptoms and assessments

#### Assessment of symptoms of ASC

To assess a comprehensive evaluation of autism symptoms in the case, we used the following standardized measures: the Autism Diagnostic Interview-Revised (ADI-R), Autism Diagnostic Observation Schedule, Second Edition (ADOS-2), Autism Behavior Checklist (ABC), Social Responsiveness Scale, and Repetitive Behavior Scale. The ADI-R is a semi-structured diagnostic interview that is typically administered to a caregiver or parent of an individual suspected of having autism (Rutter *et al*., 2003). The ADI-R results for the case exceeded the clinical cutoff for autism in all four diagnostic categories: social interaction issues (total score = 17, cutoff = 10), communication and language skills (total score = 13, cutoff = 8), repetitive and obsessive behaviors (total score = 5, cutoff = 3), and abnormality of development evident at or before 36 months (total score = 4, cutoff = 1) (Supplementary Table 1). The ADOS-2 is a semi-structured assessment administered by trained clinicians to evaluate core symptoms of ASC (Lord *et al*., 2012; Kamp-Becker *et al*., 2018). The case achieved a score of 11 on the ADOS-2 (autism spectrum cutoff = 8, autism cutoff = 9), which corresponds to a diagnosis of autism (Supplementary Table 2). The ABC is a standardized assessment that uses the observer's rating of a child's behavior to characteristics commonly associated with ASC (Volkmar *et al*., 1988; Rellini *et al*., 2004). The total score on the five scales of the ABC is employed to assess the probability of autism. The case received a total score of 47 (autism threshold = 68, questionable autism score = 53) (Supplementary Table 3). The Social Responsiveness Scale is utilized predominantly to identify and quantify social impairments associated with ASC, with scores ranging from 0 to 195 (Constantino *et al*., 2003). The case received a total score of 105 (Supplementary Table 3). The Repetitive Behavior Scale is a 44-item self-report questionnaire with six subscales that captures the scope of restricted and repetitive behavior in ASC (Bodfish *et al*., 1999). The case received a total score of 21 (Supplementary Table 3). The Individualized Psycho-educational Assessment for Children with Autism Spectrum Disorders, Third Edition, is an assessment tool used to evaluate various skills, including receptive and expressive language skills, communication abilities, and social interaction skills in children suspected of having ASC (Schopler *et al*., 2005). The results showed that the case's developmental age lagged behind the real age in all aspects (Supplementary Table 4). In summary, the case exhibited significant autism symptoms across various standardized measures.

#### Assessment of intelligence quotient

The Wechsler Intelligence Scale for Children, Fourth Edition, is a widely used intelligence test for assessing the intellectual abilities of children. The case obtained a full intelligence quotient score of 54, indicating a mental deficiency (Supplementary Table 5). Raven's Matrices is a nonverbal group test employed to evaluate abstract reasoning ability and non-verbal problem-solving skills (Raven, 2003). The case received a score of 50, indicating a mental deficiency. The Peabody Picture Vocabulary Test is a standardized assessment that gauges an individual's receptive vocabulary skills through the recognition and comprehension of spoken words, often used as an indicator of verbal intelligence quotient. The case obtained a standard score of 58. His percentile rank of 30 indicated that he scored ≥30% compared to examinees of his age. His test-age equivalent was 4:4, and his performance in receptive vocabulary comprehension fell within the extremely low range. In summary, the case exhibited mental retardation based on the intelligence quotient measures mentioned previously.

#### Assessment of arithmetic ability

The Arithmetic Test for Primary 1, was revised from the Woodcock–Johnson IV Tests of Achievement to assess the arithmetic ability of the case (Wendling, 2014). This test consists of: (i) 22 two-digit addition and subtraction problems, eight multiplication problems with single-digit numbers, and four division problems with two-digit numbers divided by one-digit numbers; and (ii) math concept questions: 16 two-digit applied addition and subtraction problems, four formula problems that change the addition formula into the multiplication formula, and four computation problems that change the multiplication formula into the addition formula (all with single-digit numbers). The case completed the test in 8 minutes and 12 seconds (time limit of 30 minutes). His percentile rank in the top 5% indicated that he scored ≥95% of examinees of his age, attaining the talented level. The results are based on a normative sample of 1569 individuals. Notably, his abilities extended beyond the simple computational problems on the test, as he proficiently solved even three-digit multiplication problems. Consequently, we randomly generated (i) 10 multiplication problems with two digits by two digits and (ii) 10 multiplication problems with three digits by three digits to evaluate his computational proficiency with more challenging problems. He completed the exam in 5 minutes with 100% accuracy on the first section and 80% on the second. These results indicated that his arithmetic abilities far surpass those of his peers.

### Gray matter volume analysis

**Figure 1:**
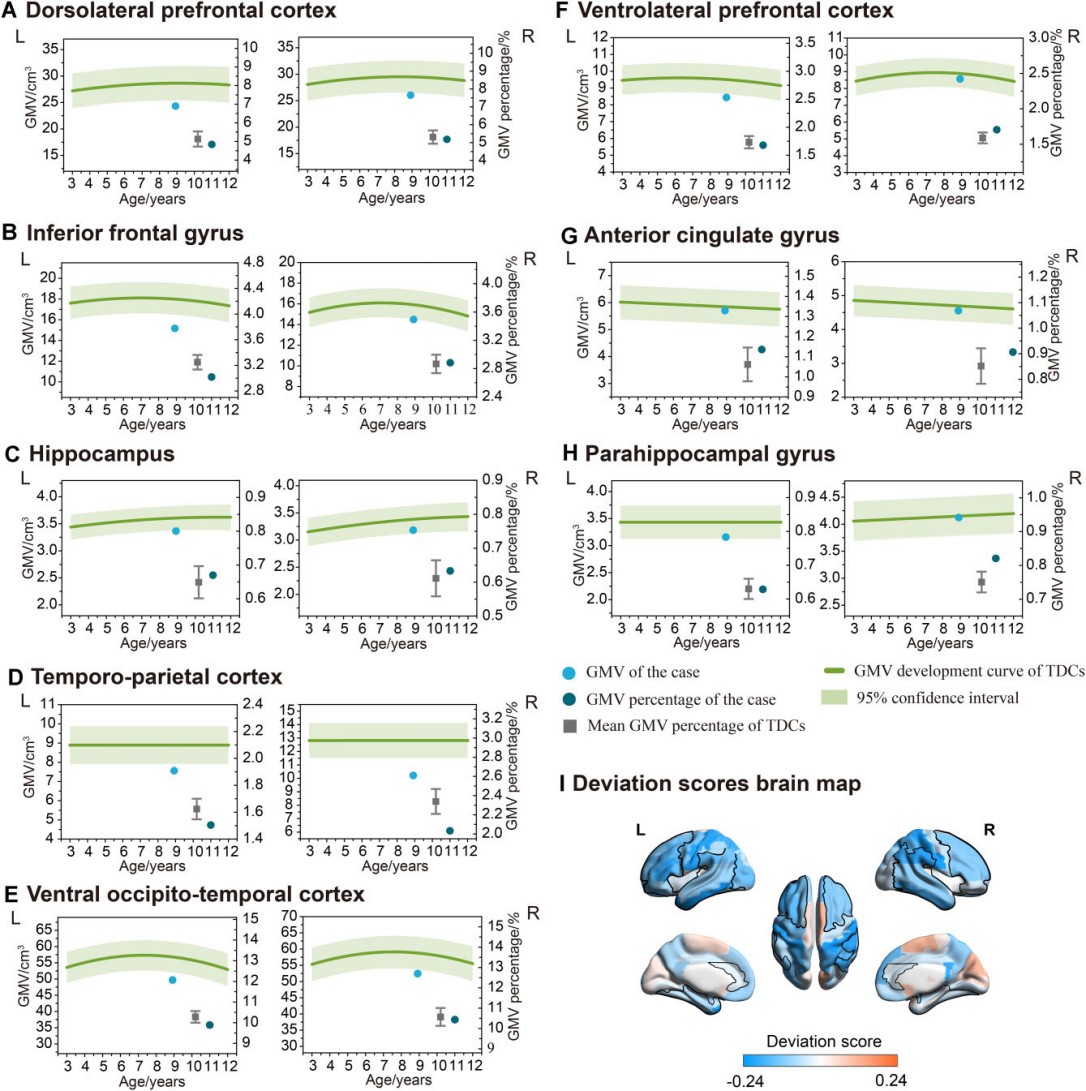
GMV comparision between the case and TDCs. (**A**)–(**H**). The gray matter developmental trajectories of arithmetic-related brain regions (top) and comparison of GMV percentages (below). The gray matter developmental trajectories were mapped from GMV of 110 TDCs using the GPR. GMV percentages were calculated using data from 17 males of comparable age, with error bars indicating a standard deviation of ±1. (**I**) Deviation scores brain map. The deviation scores were calculated based on the relative deviation of the GMV of the case from the gray matter developmental trajectories. The regions framed by the black lines correspond to brain regions associated with arithmetic. The color bar indicates the deviation scores.

We conducted structural MRI scans using a 3 Tesla GE MRI scanner. The control group consisted of 110 typically developing individuals, including 17 males of the same age as the case (as shown in Table 2). The research protocol for the discovery cohort was granted ethical approval by the University of Electronic Science and Technology of China's Research Ethics Committee. The T1-weighted images were performed using the Computation Anatomy Toolbox (CAT 12, https://neuro-jena.github.io/cat12-help/) and Statistical Parameter Mapping v.12 toolbox (SPM12, https://www.fil.ion.ucl.ac.uk/spm/software/spm12/) for VBM analysis. VBM is a neuroimaging analysis technique that entails segmentation, normalization, and smoothing stages to obtain the gray matter volume (GMV) of the brain (Ashburner & Friston, 2000). Gaussian process regression (GPR) is a probabilistic machine learning technique that models the relationship between two variables as a range of possible functions, allowing it to make predictions with uncertainty estimates (Deringer *et al*., 2021). We employed GPR to model the developmental trajectory of GMV across the age range of 3 to 12 years, using data from 110 TDCs. Age and sex were treated as independent variables, while GMV served as the dependent variable. We optimized the GPR model by adjusting the parameters to minimize the negative log marginal likelihood. Subsequently, the trained model was used to predict GMV for ages ranging from 3 to 12 years, and the developmental trajectories of arithmetic-related brain regions were visualized through plotting. The brain regions involved include the dorsolateral prefrontal cortex, ventrolateral prefrontal cortex, inferior frontal gyrus, anterior cingulate gyrus, hippocampus, parahippocampal gyrus, temporo-parietal cortex, and ventral occipito-temporal cortex. Additionally, based on the Automated Anatomical Labeling Atlas, we computed deviation scores for each brain region for the case in comparison to TDCs (Tzourio-Mazoyer *et al*., 2002). These scores were derived from the relative deviation of the case's GMV from the developmental trajectories of gray matter in typically developing children. A negative deviation score represents the case has a lower GMV in that brain region than TDCs. As shown in Fig. 1, the case exhibited reduced GMV in the bilateral dorsolateral prefrontal cortex, left ventrolateral prefrontal cortex, left inferior frontal gyrus, bilateral temporo-parietal cortex, and bilateral ventral occipito-temporal cortex. Taking into account the effect of the development of brain and whole-brain GMV, we calculated the GMV percentage in the aforementioned brain regions. Figure 1 depicts the GMV percentages of brain regions in the case and age-matched control individuals. The GMV percentages of the case were lower than those of controls in the left inferior frontal gyrus, bilateral temporo-parietal cortex, and left ventral occipito-temporal cortex. On the contrary, the GMV percentage in the left parahippocampal gyrus of the case was higher.

**Table 2:** Demographic: information of control group.

	TDC (*n* = 17)	TDC (*n* = 110)
Age range (years)	7.50–9.65	3.00–12.07
Age (years)	8.41 ± 0.66	6.48 ± 1.76
Sex (male/female)	Only male	68/42

## Discussion

This report describes an autistic child who demonstrated exceptional talent in arithmetic. The case was diagnosed with ASC accompanied by intellectual and language disorders. Gray matter analysis indicated a reduction in GMV in distinct regions within the previously mentioned arithmetic network of the case, deviating from the typical gray matter developmental trajectories observed in TDCs. The GMV percentages in the left inferior frontal gyrus, bilateral temporo-parietal cortex, and left ventrolateral prefrontal cortex were lower in the case compared to TDCs of the same age, whereas the GMV percentage of his left parahippocampal gyrus was higher. These findings indicate distinctions in arithmetic network structure between the case and typically developing individuals, which may be associated with the case's exceptional arithmetic abilities.

A volumetric study in children with dyscalculia revealed a reduction in GMV in the parahippocampal gyrus, whereas the case demonstrated the opposite (Ranpura *et al*., 2013). Studies of typical development have shown that the cortical GMV undergoes change swiftly during the initial years of life, reaching its peaks during early childhood, and then decreases with unique regional trajectories (Narvacan *et al*., 2017). Specifically, an extensive array of regions spanning the entire brain have commonly demonstrated inverted U-shaped developmental trajectories or a decline in GMV in the frontal, temporal, and parietal cortices during late childhood and adolescence (Mills *et al*., 2014). Overall, the reduction in GMV indicates the completion of a developmental process. It seems possible that the reduction in GMV in the subject was because these brain regions were developing more rapidly than in typically developing individuals. We hypothesized that the reduced GMV in the case may be the result of a unique pattern of brain development in which his brain underwent a form of hyperdevelopment, which does not exactly imply functional impairment but might instead be related to his exceptional arithmetic abilities. Lower volumes do not necessarily equate to poorer functioning, and regions with lower volumes may reflect enhanced efficiency in functioning or adaptations and resilience, which could be particularly apparent in arithmetic skills (Mills *et al*., 2014). However, additional research is needed to confirm these hypotheses.

Overall, we conducted a multifaceted and detailed behavioral assessment of the case and performed an MRI-based analysis of his brain development, contributing preliminary hypotheses that provide a detailed example within the study of such extraordinary children. However, additional research is required to substantiate our hypothesis. We intend to conduct further investigations to support our findings, and we plan to longitudinally track this case over an extended period to gain a more comprehensive understanding. Future studies could employ larger sample sizes and more multifaceted neuroimaging methods to delve deeper into the complexities of brain development in individuals with ASC and how the unique characteristics of brain development in these individuals influence their cognitive abilities and talents.

## Conclusion

In summary, this case report highlights the unique situation of a child with ASC who possesses exceptional arithmetic abilities. While displaying typical ASC symptoms, the child's arithmetic prowess has sparked interest in the brain's structural aspects. His GMV in the arithmetic network deviates from typically developing individuals. While there may be a link between this neural deviation and his talent, further research is necessary. In conclusion, the case emphasizes the diversity and complexity of ASC. While individuals with ASC may experience challenges in social and communication domains, differences in neurodevelopment may lead to a different way of processing information and learning, which may not necessarily result in impairment but may be an example of “neurodiversity” and lead to talent. Nevertheless, brain development should follow established patterns, and a better understanding of brain development in ASC would also have important implications for educational and behavioral programs.

## Supplementary Material

kkae008_Supplemental_File
