# Supplementary material for: Brain development of a school-aged boy with autism spectrum condition talented in arithmetic: a case report
Source: Psychoradiology. 2024 Apr 18;4:kkae008. doi: 10.1093/psyrad/kkae008 (PMC11074990; doi:10.1093/psyrad/kkae008)
Supplement: kkae008_Supplemental_File [file kkae008_supplemental_file.docx]

**Supplementary**

**Supplementary Table 1.** Results of the assessment of symptoms of ASC using ADI-R

| **Domains** | **Standard scores** | **Scores** |
| --- | --- | --- |
| Social interaction issues | Cutoff score: 10 | 17 |
| Communication and language skills | Cutoff score of B(Verbal): 8 | 13 |
|  | Cutoff score of B(Non-Verbal): 8 | / |
| Repetitive and obsessive behaviors | Cutoff score: 3 | 5 |
| Abnormality of development evident at or before 36 months | Cutoff score: 1 | 4 |

ASC: Autism spectrum condition; ADI-R: Autism Diagnostic Interview-Revised.

**Supplementary Table 2.** Results of the assessment of symptoms of ASC using ADOS-2

| **ADOS-2 scales** |  |  |
| --- | --- | --- |
| **Social affect (SA)** |  |  |
| Pointing | A-6 | 0 |
| Descriptive, conventional, instrumental, or, informational gestures | A-7 | 0 |
| Unusual eye contact | B-1 | 2 |
| Facial expressions directed to others | B-2 | 1 |
| Shared enjoyment in interaction | B-3 | 0 |
| Showing | B-5 | 0 |
| Spontaneous initiation of joint attention | B-6 | 0 |
| Quality of social overtures | B-8 | 1 |
| Amount of reciprocal social communication | B-11 | 0 |
| Overall quality of rapport | B-12 | 2 |
| **SA total** |  | 6 |
| **Restricted and repetitive behavior (RRB)** |  |  |
| Stereotyped/idiosyncratic use of words or phrases | A-4 | 1 |
| Unusual sensory interest in play material/person | D-1 | 1 |
| Hand and finger and other complex mannerism | D-2 | 2 |
| Unusually repetitive interests or stereotyped behaviors | D-4 | 1 |
| **RRB total** |  | 5 |
| **Overall total** |  | 11 |
| **Comparison score** |  | 6 |

ASC: Autism spectrum condition; ADOS-2: Autism Diagnostic Observation Schedule, Second edition.

**Supplementary Table 3****.** Results of the assessment of symptoms of ASC using ABC, SRS, RBS

| **Domains** | **Scores** |
| --- | --- |
| **ABC** |  |
| Sensory | 5 |
| Relating | 6 |
| Body and Object use | 9 |
| Language | 13 |
| Self-help | 14 |
| Total score | 47 |
| **SRS** |  |
| Social Awareness | 16 |
| Social Cognition | 25 |
| Social Communication | 34 |
| Social Motivation | 11 |
| Restricted Interests and Repetitive Behavior | 19 |
| Total score | 105 |
| **RBS** |  |
| Stereotypic Behavior | 5 |
| Self-Injurious Behavior Subscale | 4 |
| Compulsive Behavior Subscale | 0 |
| Ritualistic Behavior Subscale | 4 |
| Sameness Behavior Subscale | 3 |
| Restricted Interests Subscale | 5 |
| Total score | 21 |

ASC: Autism spectrum condition; ABC: Autism Behavior Checklist; SRS: Social Responsiveness Scale; RBS: Repetitive Behavior Scale.

**Supplementary Table 4.** Results of the assessment of behaviors and skills of ASC using PEP-3

| **Developing functional scale** |  |  |  |  |
| --- | --- | --- | --- | --- |
|  | **P** | **E** | **Score** | **Age Equivalent (months)** |
| Imitation | 6 | 2 | 10 | 37 |
| Perception | 11 | 0 | 11 | 60 |
| Fine motor | 5 | 5 | 10 | 36 |
| Gross motor | 9 | 2 | 11 | 41 |
| Hand-eye coordination | 12 | 2 | 14 | 62 |
| Cognitive performance | 12 | 6 | 20 | 50 |
| Cognitive verbal | 11 | 4 | 19 | 53 |
| Development total |  |  | 66 | 43 |
| **Pathology scale** |  |  |  |  |
|  | **S** | **M** | **Score** |  |
| Emotion | 1 | 4 | 6 |  |
| Interpersonal relationship | 0 | 5 | 7 |  |
| Game and object preferences | 0 | 5 | 6 |  |
| Sensory patterns | 2 | 6 | 4 |  |
| Language | 1 | 4 | 11 |  |

ASC: Autism spectrum condition; PEP-3: Individualized Psycho-educational Assessment for Children with Autism Spectrum Disorders, Third Edition; P: established capacity; E: partial capacity; M: mild; S: severe.

**Supplementary Table 5.** Results of the assessment of IQ using WISC-IV

| **Subscales** | **Scores** | **Percentile level** | **95% Confidence Interval** |
| --- | --- | --- | --- |
| Verbal Comprehension | ≤48* | <0.1 | 44-58 |
| Perceptual Reasoning | 64 | 1 | 59-75 |
| Working Memory | 67 | 1 | 62-76 |
| Processing Speed | 62 | 1 | 58-76 |
| Full Scale Intellectual Quotient | ≤54* | 0.1 | 50-60 |

IQ: Intellectual Quotient; *: The case exhibited insufficient proficiency on certain scales to be accurately assessed by this test, and the obtained results are therefore considered estimates.
